# Clinical Response to Apatinib Combined With Brain Radiotherapy in EGFR Wild-Type and ALK-Negative Lung Adenocarcinoma With Multiple Brain Metastases

**DOI:** 10.3389/fonc.2020.00517

**Published:** 2020-04-15

**Authors:** Xiaofang Ying, Huali Liu, Mingwei Wang, Min Peng, Peng Ruan, Vivek Verma, Guang Han

**Affiliations:** ^1^Department of Radiation Oncology, Hubei Cancer Hospital, Tongji Medical College, Huazhong University of Science and Technology, Wuhan, China; ^2^Department of Oncology, Renmin Hospital of Wuhan University, Wuhan, China; ^3^Department of Pathology, Hubei Cancer Hospital, Tongji Medical College, Huazhong University of Science and Technology, Wuhan, China; ^4^Department of Radiation Oncology, Allegheny General Hospital, Pittsburgh, PA, United States

**Keywords:** apatinib, EGFR wild-type, ALK-negative, lung adenocarcinoma, brain metastases

## Abstract

**Background:** Brain radiotherapy is the standard treatment option for multiple brain metastases (BMs) from non-small cell lung cancer (NSCLC), especially in the absence of a driver mutation. However, the prognosis for such patients remains poor. Apatinib is a potent antiangiogenic compound directed at the vascular endothelial growth factor receptor-2 (VEGFR-2); however, to date, there are no investigations of apatinib concurrent with brain radiotherapy for NSCLC patients with BMs. We report a case of EGFR wild-type and ALK-negative lung adenocarcinoma patient with multiple symptomatic BMs, who received apatinib together with brain radiation therapy. A favorable oncologic outcome was achieved for both brain metastatic lesions and the primary pulmonary tumor.

**Case Presentation:** A 61-year-old female (never smoker) who initially presented with headache and dizziness was diagnosed with lung adenocarcinoma with multiple brain metastasis (cT2aN3M1b stage IV), and was negative for EGFR and ALK. The patient refused to receive chemotherapy and was only amenable to brain radiotherapy and targeted therapy. After approval from the institutional ethics committee, she underwent concurrent oral apatinib (500 mg/day) with whole brain radiation therapy (WBRT) (37.5Gy) with simultaneous in-field boost (49.5Gy) in 15 fractions with image guided intensity-modulated radiotherapy. Three weeks later, neurologic symptoms entirely ceased and a partial response (PR) for the BMs with near-complete resolution of peritumoral brain edema was achieved. Chest CT performed at the same time and showed shrinkage of the lung primary with a PR. The patient suffered grade III oral mucositis one week after brain radiotherapy and refused further apatinib. At 12 months after brain radiotherapy, the brain tumors remained well controlled.

**Conclusions:** This is the first known documentation of a rapid clinical response of apatinib concurrent with brain radiotherapy in a lung adenocarcinoma patient with symptomatic multiple BMs. Apatinib combined with brain radiotherapy could be an alternative treatment option for BMs from NSCLC, especially for those without a driver mutation. Further clinical trials are required to corroborate this discovery.

## Introduction

Brain metastases (BMs) can occur frequently (22–54%) in non-small cell lung cancer (NSCLC) (1, 2) In the past, palliative care was the primary treatment for brain metastatic lesions, with radiation therapy (RT) or surgical treatment considered according to functional status as well as the number and location of metastatic lesions. Recently, several investigations have reported the efficacy of the epidermal growth factor receptor (EGFR) and anaplastic lymphoma kinase (ALK) tyrosine kinase inhibitors (TKIs) for BMs from NSCLC (3). However, for NSCLC patients with multiple BMs in the absence of a driver mutation, brain radiotherapy still remains the standard treatment option. Combining brain radiotherapy with chemotherapy or radiosensitizers has also shown disappointing efficacy (4), highlighting the need for alternative effective strategies.

For many solid tumors, hypoxia within the microenvironment can cause angiogenesis (5). In context of NSCLC BMs, neo-angiogenesis is critical for the outgrowth of macrometastasis (6). Hence, efforts to synergize radiotherapy and anti-angiogenic agents may be important, which show effects both *in vitro* and *in vivo* (7). One hypothesis for improving outcomes of NSCLC patients with multiple BMs, in the absence of a driver mutation, is to explore the potential synergy between radiotherapy and anti-angiogenic therapy.

Apatinib is a novel, small molecule tyrosine kinase inhibitor. It selectively targets vascular endothelial growth factor receptor-2 (VEGFR-2) and was approved in China as subsequent-line management for advanced gastric cancer (8). Apatinib is currently being assessed in phase II/III clinical trials for the treatment of numerous malignancies, such as gastric carcinoma, lung cancer, hepatocellular cancer, esophageal carcinoma, and colorectal cancer. However, there are few clinical evidences for the efficacy and safety of the combination of apatinib and brain radiotherapy in NSCLC patients with BMs. Herein, we report a case of a lung adenocarcinoma patient with multiple BMs, with wild-type EGFR and negative ALK status, who was treated with apatinib combined with brain radiotherapy at our institution and underwent a good response.

## Case Report

**Figure 1:**
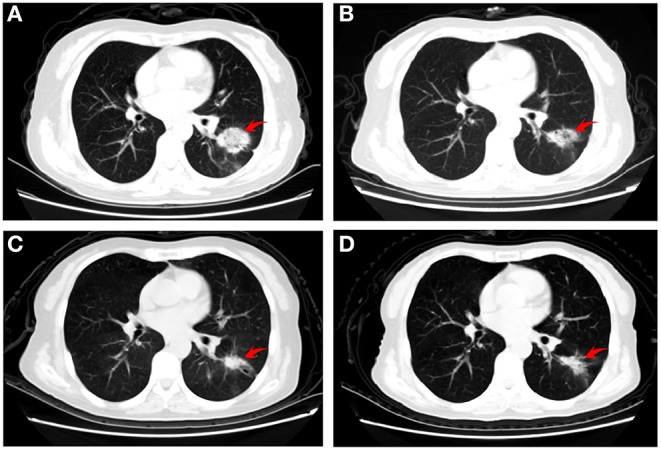
Representative computed tomography images of the patient. **(A)** baseline (before administration of apatinib) showing a left pulmonary lesion; **(B)** 3 weeks later revealing a substantial shrinkage, **(C)** 2 months after chemotherapy demonstrating an excellent tumor response; and **(D)** 4 months after chemotherapy illustrating stable disease.

**Figure 2:**
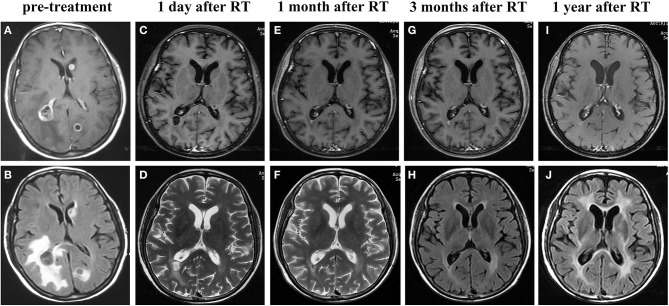
Representative magnetic resonance imaging images of the brain metastatic lesions at different time points. Prior to the treatment showing lesions in the left occipital lobe, right temporo-occipital lobe junction and a large region of edema according to enhanced T1-weighted MRI **(A)** and T2-weighted FLAIR MRI **(B)**. On the first day after finishing the whole course of brain radiotherapy, showing shrinkage of tumors in enhanced T1-weighted MRI **(C)** and T2-weighted MRI **(D)**, along with marked alleviation of cerebral edema. Enhanced T1-weighted MRI **(E,G,I)**,T2-weighted MRI **(F)** and T2-weighted FLAIR MRI **(H,J)** performed at 1, 3, 12 months after brain radiotherapy showed the brain tumors were well controlled. RT, radiotherapy.

A 61-year-old never-smoking female was admitted with the chief complaint of headache and dizziness for 2 weeks and was subsequently diagnosed with stage IV (cT2aN3M1b) lung adenocarcinoma. Chest computed tomography (CT) revealed a 3.6 × 2.8 cm left lung mass (Figure 1A) with bilateral hilar, mediastinal, and supraclavicular lymphadenopathy. Brain magnetic resonance imaging (MRI) demonstrated multiple BMs with high peritumoral brain edema (PBE) (Figures 2A,B). Lung adenocarcinoma was histologically diagnosed by excisional biopsy of a supraclavicular lymph node. No mutations were detected for EGFR or ALK.

Since the BMs were accompanied with high PBE, mannitol (or dexamethasone) was used to control the symptoms, which appeared to be ineffective. We then hypothesized that angiogenic therapy may be effective to control PBE.

The patient initially refused chemotherapy and was only amenable to cerebral radiotherapy and targeted therapy. After approval by the local ethics committee and the patient gave written informed consent, she underwent oral apatinib (500 mg/day) together with whole brain radiation therapy (WBRT) (37.5Gy) with simultaneous in-field boost (49.5Gy) in 15 fractions over three weeks with image guided intensity-modulated radiotherapy. On the first day after finishing the whole course of brain radiotherapy, the patient's neurologic symptoms entirely ceased and a partial response (PR) for the BMs with near-complete resolution of PBE was achieved (Figures 2C,D). Chest CT performed at the same time and showed shrinkage of the lung primary with a PR (Figure 1B). Hypertension, proteinuria, or hand-and-foot syndrome was not observed during apatinib treatment, but the patient suffered grade III oral mucositis (Common Terminology Criteria for Adverse Events, version 4.03) one week after brain radiotherapy and refused further apatinib. She then agreed to receive combination pemetrexed/cisplatin chemotherapy for two cycles before refusing further chemotherapy due to hyperemesis. At 2 and 4 months after chemotherapy, the thoracic disease remained stable (Figure 1) and there was a clinical complete response (CR) for the BMs (Figures 2E–H). The primary pulmonary lesion remained stable for 5 months after chemotherapy; it began to grow thereafter and the patient refused to receive further chemotherapy. Radiotherapy was thus applied to the primary lung lesion. At 12 months after brain radiotherapy, the primary lesion remained well controlled, with a tumor size of 2.5 × 1.7 cm; no BM lesions could be seen at that time. (Figures 2I,J).

**Figure 3:**
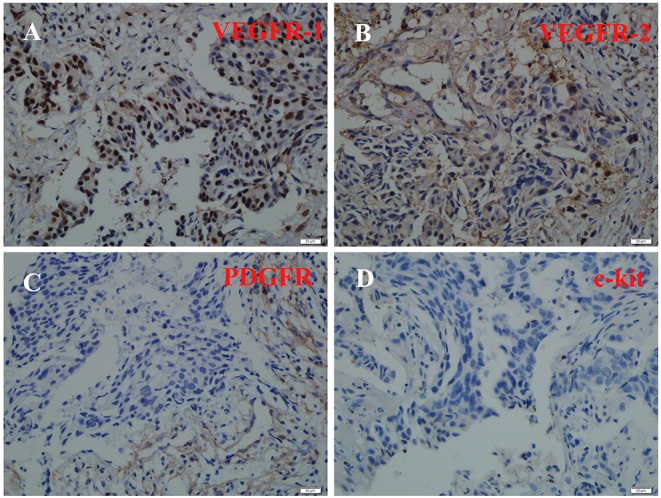
Immunohistochemical staining of VEGFR-1, VEGFR-2, PDGFR, and c-kit. VEGFR-1**(A)**, VEGFR-2 **(B)**, and PDGFR **(C)** were strongly positive, while c-kit **(D)** was negative.

Immunohistochemical staining assisted in exploring the mechanisms of the good response in this patient. The results showed that VEGFR-1, VEGFR-2, and PDGFR were strongly positive, while c-kit was negative (Figure 3).

## Discussion

In non-driver-mutated NSCLC, brain radiotherapy is a prime treatment option for multiple symptomatic BMs; however, it may not be safe to treat patients with high PBE with WBRT alone since PBE is a major cause for symptoms and death in these patients. Although effective for PBE, corticosteroid administration can lead to numerous adverse effects that can impair quality of life (9), thereby illustrating the necessity for substitute compounds.

The mechanisms of PBE are still not clear, and it is widely considered that PBE has a close relationship with tumor vascular abnormalities (10). Preclinical data of a murine BM model using repetitive multi-photon *in vivo* imaging revealed that neo-angiogenesis is critical for macrometastasis outgrowth for NSCLC BMs (7). Targeted angiogenesis can lead to regression or normalization of newly-formed vessels, reducing the vascular permeability (11), which contributes to the decrease of PBE.

Previous research showed a consistent result that bevacizumab, a humanized murine monoclonal antibody against the VEGF molecule, can decrease PBE (12). Regarding the concurrent use of bevacizumab and brain radiotherapy, there is a phase I multicentre single-arm investigation of the safety and preliminary efficacy of combination therapy for BMs from solid tumors in 19 patients (13 with breast cancer). The result showed that the combination was tolerable and appropriate; seven of eight patients had a BM response (one with CR). Further phase II trials are ongoing (13).

Apatinib is a novel orally administered small-molecule TKI, which mainly selectively competes with VEGFR-2's ATP-binding site in the cell (14). VEGFR-2, one of the VEGFR family proteins, mediates VEGF's angiogenic, mitogenic, and permeability-enhancing effects (15). Hence, inhibiting VEGFR-2 could be a novel strategy to attenuate tumor-induced angiogenesis. Several preclinical and clinical trials have proved the safety and efficacy of apatinib as a part of anti-angiogenic treatment (16, 17); it was shown that apatinib can “normalize” tumor blood vessels and reduce vascular permeability. Furthermore, apatinib may increase oxygenation by normalization of tumor vasculature and regulation of the tumor microenvironment, thus increasing overall radiosensitivity (18). Taken together, these were some factors leading to the use of apatinib in this patient, along with ease of administration and lower costs as compared with intravenous bevacizumab.

Until now, there were no published reports on concurrent apatinib and brain radiotherapy for BMs. However, apatinib monotherapy has been shown to adequately control BMs from triple-negative breast cancer (19). Apatinib has also been applied to control refractory radiation-induced brain edema (20). In summary, our case is the first to report the safety and efficacy of concurrent brain radiotherapy with apatinib, in which a CR was achieved for the BMs, along with resolution of PBE.

As mentioned above, when comparing the efficacy for the concurrent use of apatinib, bevacizumab with brain radiotherapy for brain metastatic lesions, apatinib in our case as well as bevacizumab received CR. The outcome may due to angiogenic agents, as well as the brain radiation regimens. It indicated that apatinib is a potent effective drug to control brain metastasis when combining with brain radiotherapy, which needed for further clinical trial investigations.

There are proposed mechanisms regarding apatinib's augmentation of radiotherapy. Preclinical data show that there is an interaction between angiogenesis pathways and radiation-induced damage. In a radiation experiment of tumor cells, it was found that radiation dose exposure was quantitatively correlated with VEGF level. Inhibiting the expression of VEGF can significantly inhibit the hypoxia of tumor cells and thus increase their radiation sensitivity, thereby improving the effect of radiotherapy (21). This synergistic effect has been demonstrated in a variety of tumor cell lines (7).

Another interesting discovery herein was that after 3 weeks of apatinib, there was a dramatic shrinkage of the primary lesion in the left lung. Apatinib can have substantial clinical activity in patients with metastatic NSCLC who failed initial lines of treatment (16), but this is the first report showing an excellent response to apatinib after only three weeks of first-line therapy. This may potentially be explained by the PDGFR expression of this patient's neoplasm, since apatinib also targets PDGFR, RET, and c-Kit (the latter of which was negative as per Figure 3) (22).

There are notable limitations for this case report. Any case report is a retrospective observational study with low sample size. This constitutes a relatively low level of evidence; thus, any conclusions drawn from these data are preliminary, and further clinical trial is required to corroborate this discovery.

## Conclusions

This is the first (and only) known report of a rapid clinical response of apatinib concurrent with brain radiotherapy in a lung adenocarcinoma patient with symptomatic multiple BMs. There was an effect on both the BMs as well as the primary lesion. As a result, this regimen may prove useful as a treatment option for metastatic NSCLC, especially for patients without driver mutations. However, clinical trials are needed to further investigate the discovery, to elucidate the mechanisms underlying this synergy, and to screen appropriate NSCLC patients who may benefit from apatinib.

## Data Availability Statement

All datasets generated for this study are included in the article/supplementary material.

## Ethics Statement

The studies involving human participants were reviewed and approved by Ethics Committee of the Hubei Cancer Hospital, Tongji Medical College, Huazhong University of Science and Technology as well as Renmin Hospital of Wuhan University. The patients/participants provided their written informed consent to participate in this study. Written informed consent was obtained from the individual(s) for the publication of any potentially identifiable images or data included in this article.

## Author Contributions

GH conceived the idea, carried out critical interpretations of this study, and contributed to the final version of the manuscript. XY and HL collected and analyzed the data, wrote, and edited the manuscript. MW carried out the immunohistochemistry analysis and edit the manuscript. MP analyzed and interpreted the CT and MRI data. PR was responsible for clinical management. VV edited and critically revised the manuscript.

### Conflict of Interest

The authors declare that the research was conducted in the absence of any commercial or financial relationships that could be construed as a potential conflict of interest.
